# Remarkably High Hole Mobility Metal-Oxide Thin-Film Transistors

**DOI:** 10.1038/s41598-017-17066-x

**Published:** 2018-01-17

**Authors:** Cheng Wei Shih, Albert Chin, Chun Fu Lu, Wei Fang Su

**Affiliations:** 1 Department of Electronics Engineering, National Chiao Tung University, Hsinchu, 300 Taiwan; 2 Depatment of Materials Science & Engineering, National Taiwan University, Taipei, 10617 Taiwan

## Abstract

High performance p-type thin-film transistor (p-TFT) was realized by a simple process of reactive sputtering from a tin (Sn) target under oxygen ambient, where remarkably high field-effect mobility (*μ*_*FE*_) of 7.6 cm^2^/Vs, 140 mV/dec subthreshold slope, and 3 × 10^4^ on-current/off-current were measured. In sharp contrast, the SnO formed by direct sputtering from a SnO target showed much degraded *μ*_*FE*_, because of the limited low process temperature of SnO and sputtering damage. From the first principle quantum-mechanical calculation, the high hole *μ*_*FE*_ of SnO p-TFT is due to its considerably unique merit of the small effective mass and single hole band without the heavy hole band. The high performance p-TFTs are the enabling technology for future ultra-low-power complementary-logic circuits on display and three-dimensional brain-mimicking integrated circuits.

## Introduction

The metal-oxide thin-film transistors (TFTs)^1–22^ have attracted much attention for next-generation display due to its high mobility in comparison to the silicon-based TFTs, good optical transparency in visible light region, and compatibility with low-temperature processes. To incorporate control integrated circuit (IC) into display and lower the power consumption, high mobility metal-oxide p-type TFT (p-TFT) is required. Such complementary n- and p-TFTs are the needed technology for tens of years since the TFT invention^17–23^. However, most metal-oxide TFTs^1–13^ show n-type conduction. Only very few oxides such as Cu_x_O^14,18^, NiO_x_^15,16^, and SnO^20^ exhibit p-type conduction with a low mobility. Therefore, the development of high mobility metal-oxide p-TFT is crucial to embed low-power complementary logic circuits on display for system-on-panel. Previously, we pioneered very high mobility SnO_2_ n-TFTs^10–12^. In this paper, we investigated the device performance and material property of SnO p-TFT with the same Sn material. Using hafnium oxide (HfO_2_) as the gate dielectric, the HfO_2_/SnO p-TFT has a high field-effect mobility (*μ*_*FE*_) of 7.6 cm^2^/Vs, small 140 mV/dec subthreshold slope (*SS*), and 3 × 10^4^ on-current/off-current (*I*_*ON*_/*I*_*OFF*_). From the first principle quantum-mechanical calculation, the SnO is one of the best candidates for p-TFT, due to its smaller hole effective mass and unique merit without heavy hole band. The high device performance, simple process, and low-cost material make SnO the excellent candidate for future p-TFTs.

## Results

**Figure 1:**
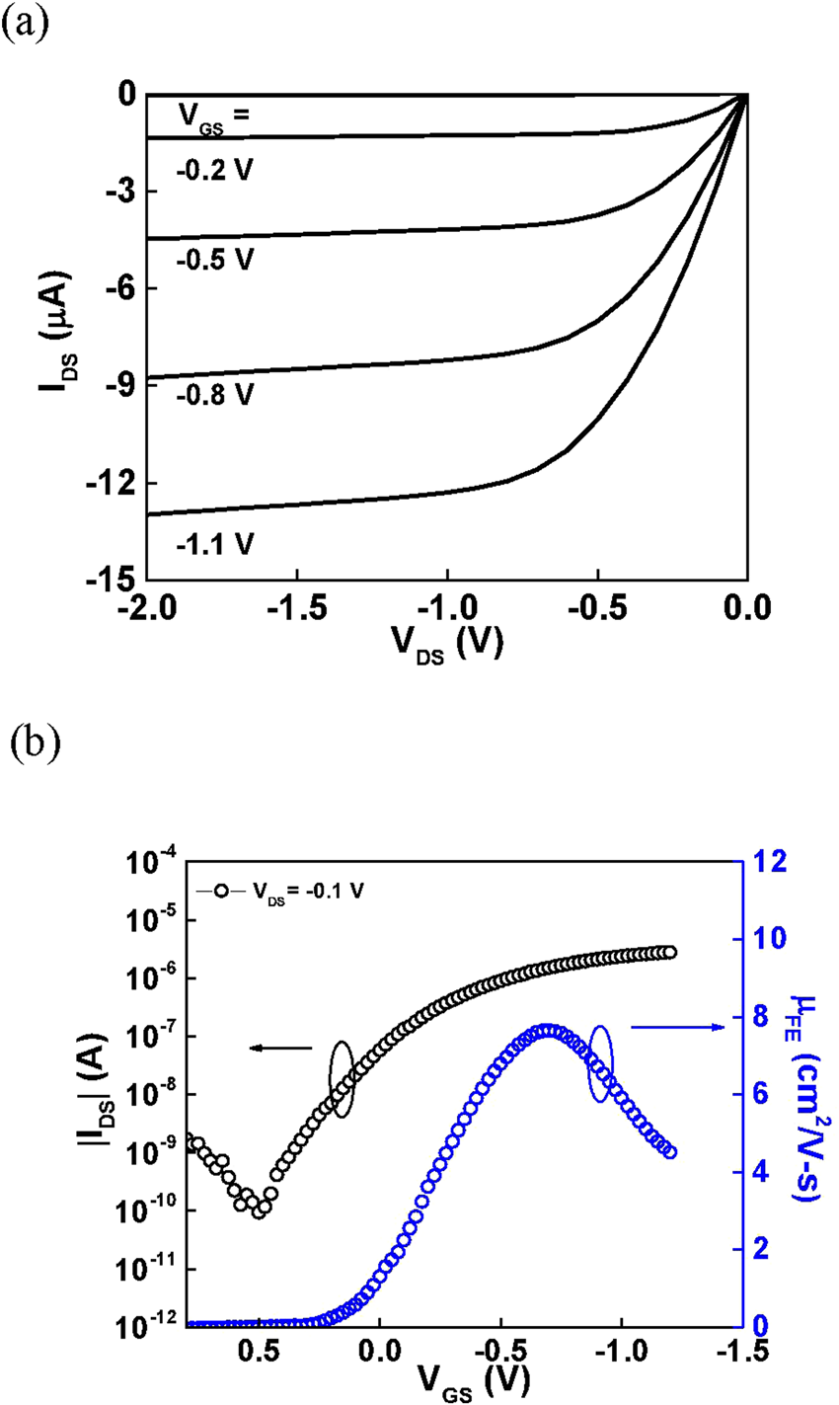
(**a**) *I*_*DS*_*-V*_*DS*_ characteristics and (**b**) |*I*_*DS*_|*-V*_*GS*_ and *μ*_*FE*_*-V*_*GS*_ characteristics of Ni/SnO_x_/HfO_2_/TaN TFTs under an Ar/O_2_ = 1.0, where the SnO_x_ was formed by reactive sputter from a Sn target.

Figure 1(a) and (b) show the transistor’s drain-source current versus drain-source voltage (*I*_*DS*_-*V*_*DS*_), |*I*_*DS*_| versus gate-source voltage (|*I*_*DS*_|-*V*_*GS*_) and *μ*_*FE*_-*V*_*GS*_ characteristics of the HfO_2_/SnO_x_ p-TFTs, where the SnO_x_ was formed by reactive sputter from a Sn target. Good device performance was reached at a low *V*_*DS*_ of −1.2 V that is vital to lower the switching power of *CV*_*DS*_^2^*f*/2, where *C* and *f* are the capacitance and operation frequency, respectively. Besides, high hole *μ*_*FE*_ of 7.6 cm^2^/Vs, a *SS* of 140 mV/dec, and an *I*_*ON*_/*I*_*OFF*_ of 3 × 10^4^ were obtained. The device mobility is among the best reported p-TFTs in literature^19,20^. It is important to notice that the device performance is highly related to oxygen content. The *μ*_*FE*_ was degraded by an order of magnitude at higher O_2_/Ar ratio, where the degraded mobility is related to the increasing SnO_2_ content inside the SnO.

**Figure 2:**
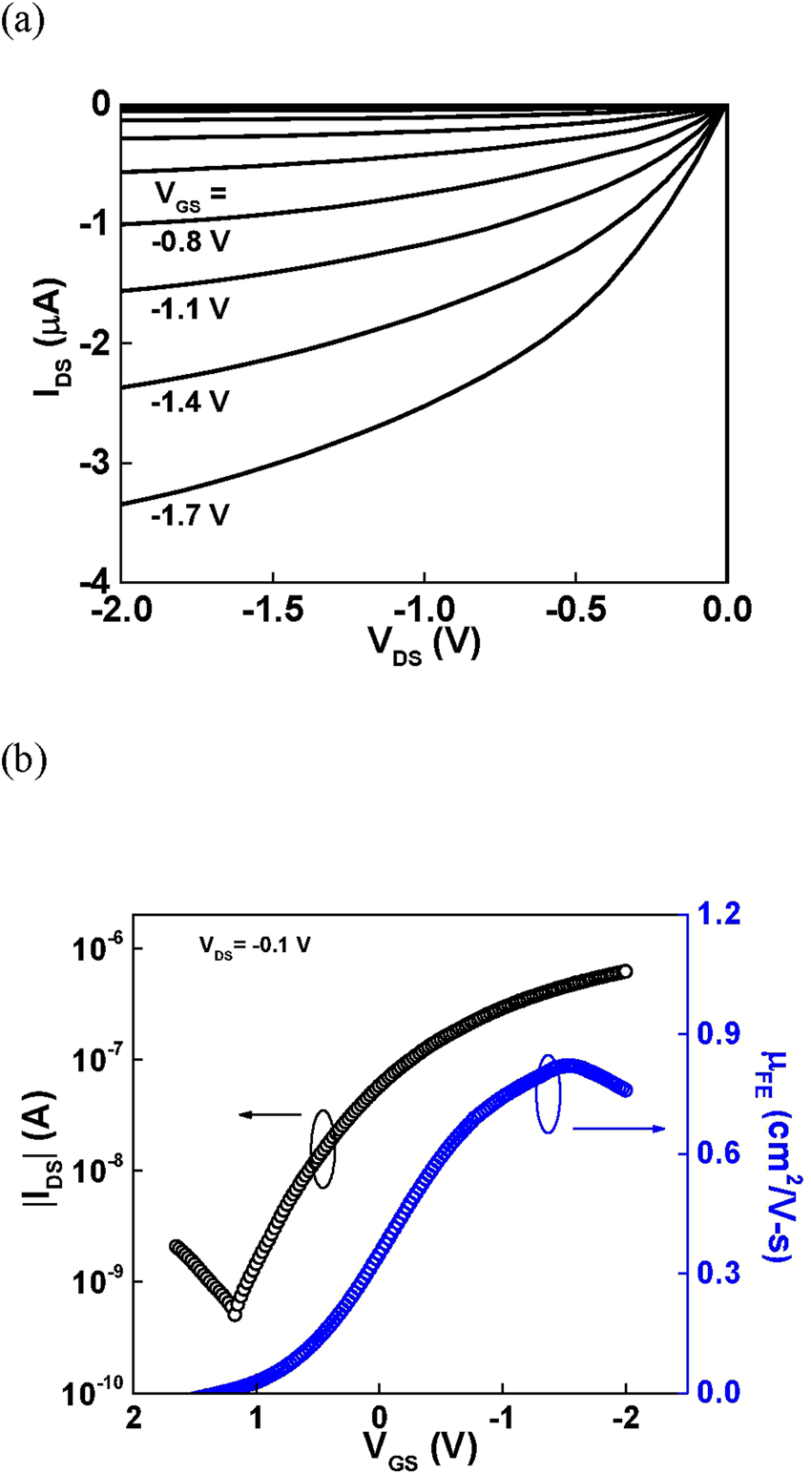
(**a**) *I*_*DS*_*-V*_*DS*_ characteristics and (**b**) |*I*_*DS*_|*-V*_*GS*_ and *μ*_*FE*_*-V*_*GS*_ characteristics of Ni/SnO_x_/HfO_2_/TaN TFTs, where the SnO_x_ was formed by sputter directly from a SnO target.

The reactive sputtering from a Sn target is the crucial technique to reach high hole mobility. Figure 2(a) and (b) show the device characteristics of HfO_2_/SnO_x_ p-TFTs, where the SnO_x_ was formed by directly sputtering from a SnO target. A low hole *μ*_*FE*_ of 0.83 cm^2^/Vs, a poor *SS* of 430 mV/dec, and a small *I*_*ON*_/*I*_*OFF*_ of 1.2 × 10^3^ were measured. Even poor *μ*_*FE*_ value was measured at annealing temperature higher than 200 °C. The high temperature is needed to anneal out the sputtering damage by energetic ions. But the annealing temperature higher than 200 °C cannot be applied to SnO device, because the SnO will translate to low mobility Sn_3_O_4_ and SnO_2_ at high temperatures^24,25^.

**Figure 3:**
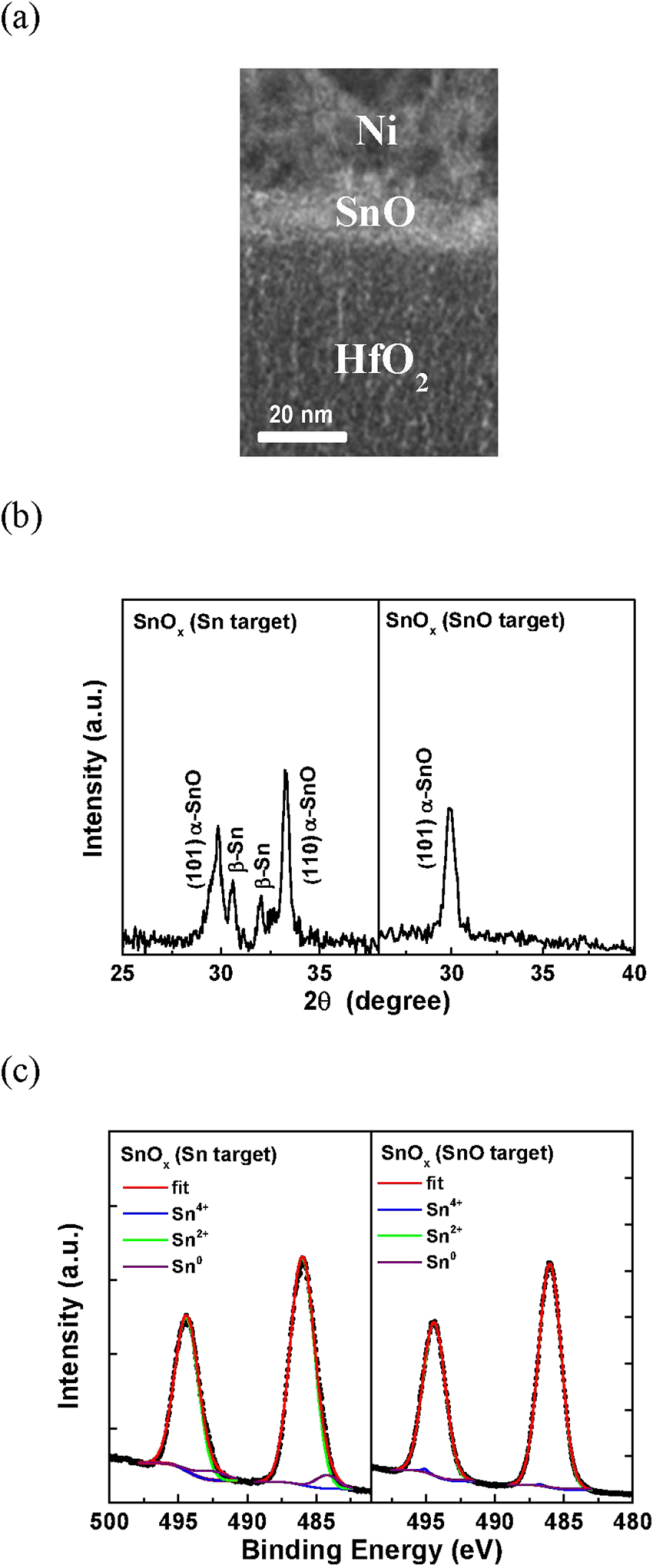
(**a**) TEM, (**b**) XRD, and (**c**) The Sn 3d XPS analysis of SnO_x_ formed from Sn and SnO targets.

We have further performed the material analysis to understand the large device performance difference between sputtering from the Sn and SnO targets. Figure 3(a) shows the structure of fabricated Ni/SnO_x_/HfO_2_ device. From the cross-sectional transmission electron microscopy (TEM), the SnO_x_ active layer on HfO_2_ has a thickness of 12 nm. The microscopic structure of SnO_x_ was analyzed by X-ray diffraction (XRD) as shown in Fig. 3(b). For SnO_x_ formed by reactive sputtering from a Sn target, a mixture of major tetragonal *α*-SnO phase and small amount of *β*-Sn phase is observed that was caused by the incomplete Sn oxidation^26,27^. In contrast, only a pure *α*-SnO phase was found from the SnO target. The atomic composition of SnO_x_ in p-TFT are further characterized by X-ray photoelectron spectroscopy (XPS) in Fig. 3(c). The de-convoluted spectra in both cases show a major Sn^2+^ peak with tiny Sn^4+^ and Sn° peaks, although the later ones are smaller for directly sputtered SnO than those from reactive sputtering of a Sn target. Therefore, the SnO_x_ by sputtering from a SnO target gives better material quality. Nevertheless, the *μ*_*FE*_ is significantly lower than that from the reactive Sn target. The potential reason may be related to the sputter damage from the SnO target, which is difficult to be detected by XRD and XPS analysis. Unfortunately such damage cannot be annealed out because of the limited process temperature of SnO, which can react as scattering centers to low the mobility. The other possibility to reach high mobility may be related to the multi-phonon assisted tunneling^28^ via small amount of metallic Sn in SnO. This is also associated with the lower off-current in reactive sputtered SnO device than that formed by sputtering from the SnO target. Further theoretical analysis will be required to understand the role of metallic Sn inside SnO. Nevertheless, the metallic Sn is difficult to form by sputtering from the SnO target.

**Figure 4:**
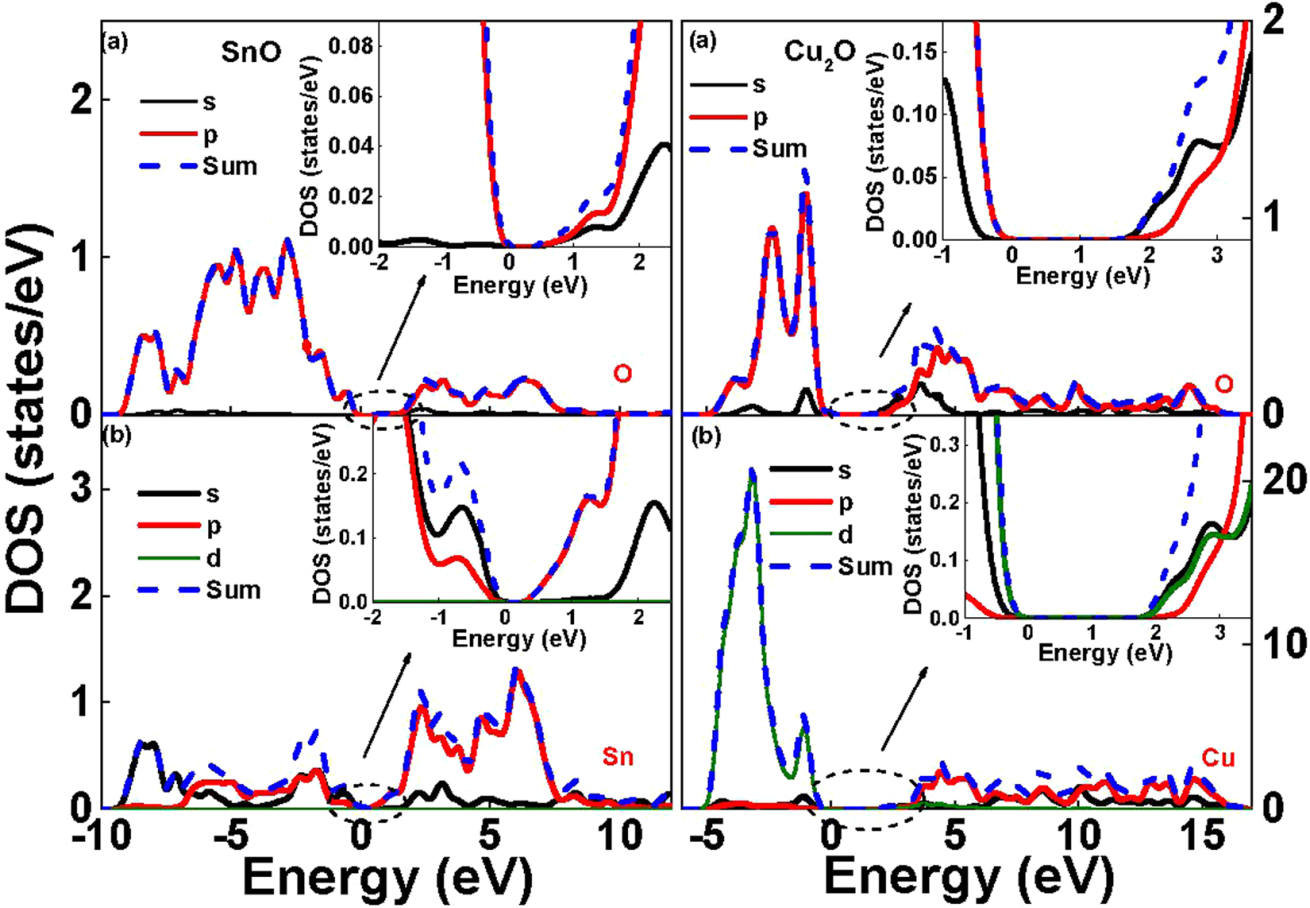
DOS distribution of SnO and Cu_2_O, where d and s, p states hybridization is found for Cu_2_O.

It is crucial to notice that the measured hole *μ*_*FE*_ is the highest value among oxide semiconductors. We further perform the first principle quantum-mechanical calculations on SnO and the other potential candidate of Cu_2_O (Figures S1 and S2). The structures of both SnO and Cu_2_O semiconductors were obtained using local density approximation plus *U* (LDA + *U*) method with appropriate *U*^*p*^ and *U*^*d*^ value. The good accuracy is supported by the calculated band structure of Cu_2_O; a direct 2.1 eV bandgap and cubic structure were obtained, agreeing well with experiments^14,29,30^. Both light hole and heavy hole bands were found in Cu_2_O that are typical for most major semiconductors of Si, Ge, GaAs, InP, InAs etc. Besides, the density of state (DOS) of heavy hole band is considerably higher than that of light hole band to cause the low hole mobility. In sharp contrast, the SnO only has a single hole band, leading to the high hole *μ*_*FE*_. The calculated DOS of SnO and Cu_2_O are further shown in Fig. 4. The Cu_2_O has much higher DOS than SnO due to its heavy hole bands. For Cu_2_O, the d-orbital holes have complex intra-atomic hybridization between d and s, p states that lowers the hole mobility. This is also applied for most oxide semiconductors to result in a low hole mobility^31–33^. In sharp contrast, the Sn 5s orbital is occupied and exhibits a s-p coupling with the O 2p ligand orbitals, unlike the p-d interaction of the d^10^ Cu_2_O. The delocalized character of the 5s states leads to a strong valence band dispersion and small hole effective masses in SnO (Figure S3). The SnO has much smaller hole effective mass than Cu_2_O and other major semiconductors of Si, Ge, GaAs, InP, InGaAs etc. This is the extremely unique merit of SnO p-type transistor to reach high hole mobility.

Table 1 compares the device performance of various TFTs. For p-TFTs, the SnO device has good hole *μ*_*FE*_, *SS*, and *I*_*ON*_/*I*_*OFF*_, which is supported from the small effective mass and single band without heavy hole band. The low *V*_*D*_ operation is important to lower AC power consumption.

**Table 1:** The device performance of various TFTs.

Channel Materials	Channel layer thickness (nm)	Gate Insulator Materials	*SS* (V/decade)	μ_EF_ (cm^2^/V·s)	I_ON_/I_OFF_	Operating Voltage (V)
NiO [15]	30	SiO_2_	—	5.2	~10^3^	−100
Cu_x_O [19]	—	ScO_x_	~0.4	0.8	~10^5^	−3
SnO [20]	15	SiO_2_	0.55	3.3	10^4^	−3
SnO [25]	27	SiO_2_	0.24	1.4	~10^4^	−100
This Work SnO	12	HfO_2_	0.14	7.6	3 × 10^4^	−1.2

In conclusion, record high hole mobility of SnO p-TFT was realized. The superb device performance, simple process, and low-cost material make SnO the excellent candidate for next generation ultra-low power display devices and 3D brain-mimicking IC^10,13^.

## Methods

The bottom-gate TFTs were fabricated on a 500-nm-thick SiO_2_ layer over a Si substrate. A 60-nm TaN was first deposited through reactive sputtering and patterned as the bottom gate electrode. Subsequently, gate dielectric of 40-nm-thick high-κ HfO_2_ was deposited through physical vapor deposition (PVD) and annealed at 400 °C. Then a 12 nm SnO_x_ was deposited by sputtering in an Ar/O_2_ mixture from a Sn target, under a DC power of 50 W and a pressure of 7.6 mTorr. Alternatively, the SnO_x_ was deposited by RF sputtering from a SnO_x_ target in the Ar/O_2_ ambient, under a power of 200 W and a pressure of 7.6 mTorr. Then both the SnO films were subjected to post-deposition annealing at 200 °C in N_2_ ambient. Finally, the Ni was deposited to form source-drain electrodes. The gate length and width were 50 and 500 μm, respectively. The electrical characteristics of the fabricated devices were measured using an HP4155B parameter analyzer and a probe station. The SnO film was analyzed by transmission electron microscopy (TEM), X-ray diffraction (XRD), and X-ray photoelectron spectroscopy (XPS). The XPS spectra were measured with a PHI 5000 VersaProbe system (ULVAC-PHI, Chigasaki) using a microfocused (100 µm, 25 W) Al X-ray beam. Cross section TEM images of devices were obtained from high resolution transmission electron microscope (JEOL 2010F, USA). The first principle quantum-mechanical calculation was applied to compute the theoretical structural and electrical properties of SnO and Cu_2_O, using Cambridge Sequential Total Energy Package code (Materials Studio 8.0) with generalized gradient approximation (GGA) and local-density approximations plus Hubbard potential *U* (LDA + *U*) method.

## Electronic supplementary material

Supplementary Information
