# Supplementary material for: Remarkably High Hole Mobility Metal-Oxide Thin-Film Transistors
Source: Sci Rep. 2018 Jan 17;8:889. doi: 10.1038/s41598-017-17066-x (PMC5772488; doi:10.1038/s41598-017-17066-x)
Supplement: Supplementary file 1 — Supplementary Information [file 41598_2017_17066_MOESM1_ESM.pdf]

Remarkably High Hole Mobility Metal-Oxide Thin-Film Transistors

Cheng W. Shih[1], Albert Chin[1*], Chun F. Lu[2], and Wei F. Su[2]

[1]Department of Electronics Engineering, National Chiao Tung University, Hsinchu 300, Taiwan

[2]Depatment of Materials Science & Engineering National Taiwan University, Taipei 10617, Taiwan

*Correspondence: Albert Chin (email: achin@faculty.nctu.edu.tw)

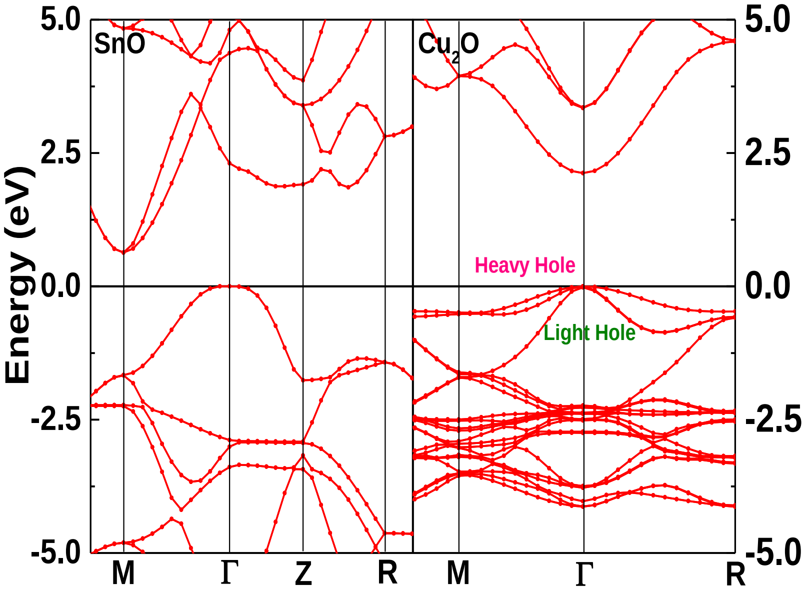

Figure S1. Band structure of SnO and Cu_2O calculated by GGA function with correction by using LDA+U.

Supplementary Figures

(a)

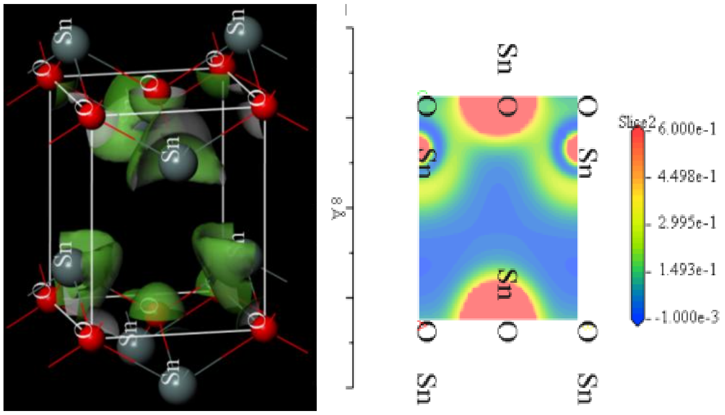

(b)

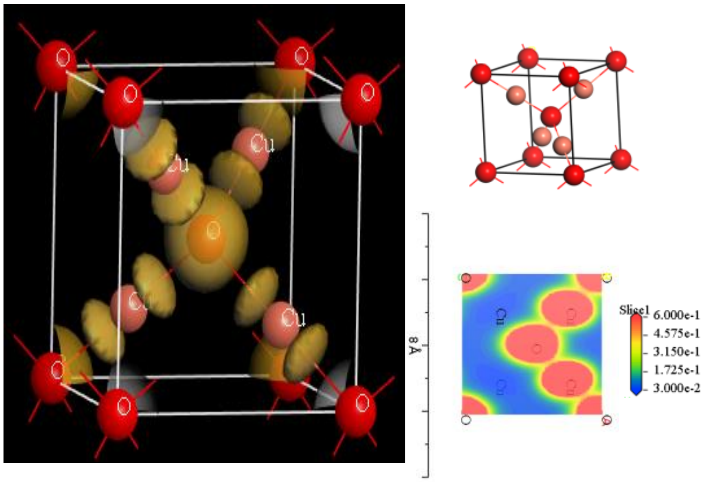

Figure S2. Charge density distribution of (a) SnO and (b) Cu_2O.

SnO		Cu_2O	
	m_h^*		m_h^{LH*}
ΓM	$-0.19\ m_0$	ΓM	$-0.65\ m_0$
ΓZ	$-0.17\ m_0$	ΓR	$-0.65\ m_0$
ΓX	$-0.23\ m_0$	ΓX	$-0.65\ m_0$

Figure S3. Hole effective mass of SnO and Cu_2O at different directions.
